# Pacific Walrus and climate change: observations and predictions

**DOI:** 10.1002/ece3.317

**Published:** 2012-07-22

**Authors:** James G MacCracken

**Affiliations:** U.S. Fish and Wildlife Service, Marine Mammals Management1011 E. Tudor Rd, MS-341, Anchorage, Alaska, 99503

**Keywords:** Adaptation, habitat change, mitigation, *Odobenus rosmarus divergens*, scenario analyses, subsistence harvest and culture

## Abstract

The extent and duration of sea-ice habitats used by Pacific walrus (*Odobenus rosmarus divergens*) are diminishing resulting in altered walrus behavior, mortality, and distribution. I document changes that have occurred over the past several decades and make predictions to the end of the 21st century. Climate models project that sea ice will monotonically decline resulting in more ice-free summers of longer duration. Several stressors that may impact walruses are directly influenced by sea ice. How these stressors materialize were modeled as most likely-case, worst-case, and best-case scenarios for the mid- and late-21st century, resulting in four comprehensive working hypotheses that can help identify and prioritize management and research projects, identify comprehensive mitigation actions, and guide monitoring programs to track future developments and adjust programs as needed. In the short term, the most plausible hypotheses predict a continuing northward shift in walrus distribution, increasing use of coastal haulouts in summer and fall, and a population reduction set by the carrying capacity of the near shore environment and subsistence hunting. Alternatively, under worst-case conditions, the population will decline to a level where the probability of extinction is high. In the long term, walrus may seasonally abandon the Bering and Chukchi Seas for sea-ice refugia to the northwest and northeast, ocean warming and pH decline alter walrus food resources, and subsistence hunting exacerbates a large population decline. However, conditions that reverse current trends in sea ice loss cannot be ruled out. Which hypothesis comes to fruition depends on how the stressors develop and the success of mitigation measures. Best-case scenarios indicate that successful mitigation of unsustainable harvests and terrestrial haulout-related mortalities can be effective. Management and research should focus on monitoring, elucidating effects, and mitigation, while ultimately, reductions in greenhouse gas emissions are needed to reduce sea-ice habitat losses.

## Introduction

Arctic and subarctic regions are disproportionately affected by current climate warming that appears to be driven by anthropogenic greenhouse gas emissions (Arctic Climate Impact Assessment [ACIA] 2005; Intergovernmental Panel on Climate Change [IPCC] 2007; Blunden et al. 2011). Predicting how species will respond to climate change is a priority for researchers, managers, and policy makers (National Research Council of the National Academies [NRC] 2009; Fleishman et al. 2011). Several assessments of the effects of a warming climate on arctic ecosystems and species have been made (ACIA 2005; Arctic Monitoring and Assessment Programme [AMAP] 2009; Huntington 2009; Wassmann et al. 2011), but have largely dealt with generalities. However, Laidre et al. (2008) assessed the sensitivity of many arctic marine mammals to climate-induced habitat changes associated with changes in sea-ice dynamics. In addition, Hovelsrud et al. (2008) examined human–marine mammal interactions, with a focus on subsistence harvest, and Metcalf and Robards (2008) and Robards et al. (2009) examined human–Pacific walrus (*Odobenus rosmarus divergens*) interactions specifically. All these assessments noted a lack of baseline as well as current data for many marine species, ecosystems, and processes that would be useful in assessing climate change effects.

Population and community dynamics are difficult to predict and “ecological surprises” appear to be the norm (Doak et al. 2008). Because predictions are often based on current conditions and the recent-past, a lack of baseline information is problematic (Kass et al. 2011) and predictions become even more uncertain when no analog situations arise, as may occur under some climate change scenarios (Williams and Jackson 2007). The future is uncertain and several realistic scenarios can be formulated (Gray 2011). Limiting analyses to a subset of scenarios that exclude other plausible outcomes is unwise and potentially misleading; each scenario should be treated as a working hypothesis (Chamberlin 1890) until it can be proven false.

Several marine mammals are adapted to arctic sea-ice dynamics (Jefferson et al. 2008; Kelly 2010), particularly polar bears, walrus, and the “ice seals” (ringed [*Pusa hispida*], ribbon [*Histriophoca fasciata*]*,* spotted [*Phoca larga*], and bearded [*Erignathus barbatus*]). As the seasonal dynamics of ice cover on arctic seas change, many marine mammals are being affected to various degrees (Laidre et al. 2008). Laidre et al.'s (2008) sensitivity index resulted in walrus being ranked as moderately sensitive to climate-induced habitat change, but noted that the Pacific subspecies would likely be impacted to a greater extent than the Atlantic walrus (*O. r. rosmarus*).

The purposes of this article are to (1) describe environmental changes over the last 20–30 years, how those changes influence a number of stressors, and walrus responses to those changes; and (2) predict how those stressors will develop and how walrus will respond out to the end of the 21st century, after a brief review of Pacific walrus ecology and behavior. Because the future intensity of many stressors is dependent on economic forces, policy responses, and the efficacy of mitigation programs, we integrate the results of a scenario analyses outlined in MacCracken et al. (2012) into four working hypotheses which can be used to identify emerging trends, predict outcomes, and identify the appropriate management responses and research needs.

## Pacific Walrus Ecology and Behavior

**Figure 1:**
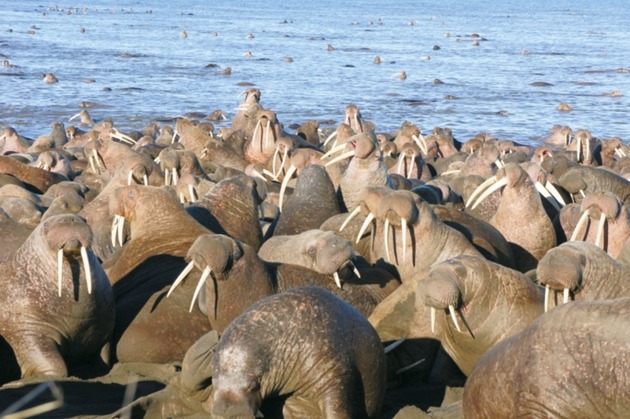
Photograph of a group of adult female Pacific walruses and younger animals resting on shore near the Native Village of Point Lay, Alaska. (Photo credit: Bill Tracey).

Pacific walrus are closely associated with the annual formation and melting of sea ice. Walrus feed primarily on benthic invertebrates (bivalves, gastropods, polychaetes) and rest on sea ice or shore (Fig. 1) between feeding dives (Fay 1982; Fay et al. 1984; Jefferson et al. 2008). The entire population winters in the Bering Sea in broken pack ice where breeding typically occurs in January–February (Ray and Watkins 1975; Fay 1982). As the ice retreats to the north in the spring, the majority of females and dependent young (newborns to 3-year olds) follow the ice edge north into the Chukchi Sea. Walruses have a prolonged gestation, and birth of offspring conceived the previous year occurs on the ice before and during the spring migration. Sea ice also serves as a substrate for nurturing young, shelter from storms, cover and isolation from predators, passive transportation, and plays a role in their lek-like breeding behavior (Ray and Watkins 1975; Fay 1982; Jefferson et al. 2008).

Many adult male walruses remain in the Bering Sea during summer and forage from haulouts along the mainland coast of the Russian Federation and United States (Fay et al. 1984). These walruses tend to use summer haulout sites repeatedly and exhibit fidelity to those sites (Jay and Hills 2005). However, haulout use is dynamic with numbers of animals fluctuating daily and the abandonment and formation of haulouts is likely dictated by prey availability, human disturbance, coastal development, etc. (Jay and Hills 2005). Herd size at haulouts can exceed 100,000 animals.

Walruses on terrestrial haulouts can be disturbed by predators, aircraft, boats, tourists/recreationists, hunters, feral dogs, etc. (Jay et al. 2011). When threatened, walrus flee to the ocean *en mass* which can lead to trampling and death of conspecifics, particularly smaller animals. Disturbance-related stampedes can result in the death of hundreds to thousands of animals each year (Fay and Kelly 1980; Fischbach et al. 2009; Jay et al. 2011). In addition, there is a background level of haulout-associated mortality among younger animals due to normal herd behaviors such as threat displays, fighting among bulls, maneuvering for preferred positions within a herd, and general agonistic behavior (Fay 1982).

In contrast, female walruses, particularly those with dependent young, do not use coastal haulouts to as great an extent. They are relatively limited in the distances they can travel to find prey, due primarily to young in tow, and they probably avoid using terrestrial haulouts to reduce mortalities of young. Walruses that summer in the Chukchi Sea occupy the broken pack ice along or near the ice edge (Ray et al. 2010) providing access to thousands of km^2^ of the benthic zone that has a high abundance of invertebrate prey (Grebmeier et al. 1989; Sirenko and Gagaev 2007). This strategy allows for efficient foraging and maximizes the survival of young (Fay 1982).

Estimates of population size for periods prior to 1975 were extrapolated from trends in harvest levels (Fay et al. 1989, 1997; Table 1). Between 1975 and 1990, aerial surveys were carried out by the United States and Russia at 5-year intervals, producing population estimates ranging from 201,000 to 246,000 (Table 1). The program was suspended in 1990 due to costs and the imprecision of the estimates. Technical advances that allowed for detectability corrections, and management needs prompted another aerial survey in April 2006, but the resulting point estimate was biased low and the confidence interval spanned an order of magnitude (Speckman et al. 2011; Table 1). Aerial survey results are not directly comparable among years due to differences in survey methods, timing of surveys, segments of the population surveyed, and incomplete coverage of areas where walruses may have been present (Fay et al. 1997); and do not provide a reliable estimate of population trend (Hills and Gilbert 1994; Gilbert 1999).

**Table 1:** Estimates of the harvest and population size of Pacific walrus

Time period	Annual harvest	Population size
1650–1790	5000–6000^1^	No estimate
Early 1800s	10,000^2^	No estimate
1860–1872	12,000–60,000^1^,^3^	No estimate
1885–1914	100^4^	80,000^1^
1915–1950	5000–7000^1^	50,000–100,000^5^
1960–1969	5300 (512)^6^	75,400–159,600^5^
1970–1979^7^	5700 (377)^6^,^8^	221,000 (–20,000 to 480,000)^9^,^10^
1980–1984	11,000 (837)^6^	246,000 (–20,000 to 540,000)^10^,^11^,^12^
1985–1989	10,896 (1378)^6^	234,020 (–20,000 to 510,000)^10^
1990–1999	6307 (707)^6^	201,000 (–19,000 to 460,00)^10^,^13^
2000–2009	5410 (511)^6^	129,000 (55,000–550,000)^14^

1 Fay (1957).

2 Elliot (1882).

3 Scammon (1874).

4 Bockstoce and Botkin (1982).

5 Fay et al. (1997).

6 U. S. Fish and Wildlife Service, unpublished data. Annual mean (standard error [SE]) for the decade.

7 From 1975 to 1990, joint United States and Russian aerial population surveys were conducted at 5-year intervals.

8 Alaska had a 3000 animal quota from 1976 to 1979.

9 Gol'tsev 1976, Estes and Gilbert 1978, Estes and Gol'tsev 1975, Udevitz et al. (2001).

10 95% confidence interval, in parentheses, from Hills and Gilbert (1994).

11 Johnson et al. (1982).

12 Fedoseev (1984).

13 Gilbert et al. (1992).

14 Speckman et al. (2011).

**Figure 2:**
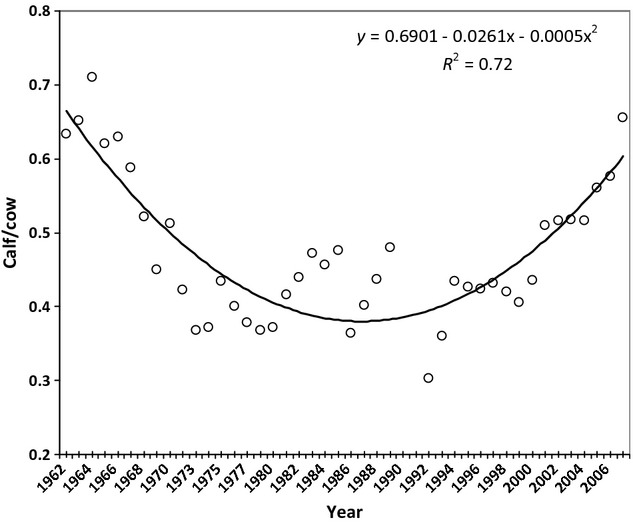
Five-year moving average of calf/cow estimates of Pacific walrus based on harvest returns to St. Lawrence Island from 1960 to 2010. Data for 1990 and 1991 is unavailable. Trend line is from an ordinary least squares regression, presented above the line.

The Bering and Chukchi Seas appear to have had a carrying capacity (K) of 250,000–300,000 + walruses based on population estimates (Table 1), and prey abundance and intake rates presented by Fay (1982). The status of the population relative to K of the region has been inferred largely from harvest data, field observations, and population surveys (United States Fish and Wildlife Service [USFWS] 2011). The working hypothesis is that: (1) hunting prior to 1960 kept the population well below K, (2) the establishment of harvest regulations resulted in a population increase that peaked in the 1980s close to or at K (Fay et al. 1989, 1997), (3) a subsequent decline in productivity and increases in harvest reduced the population, and (4) the population has remained below K to the present largely due to subsistence hunting. Trends in reproductive rates of harvested females are consistent with this scenario (Garlich-Miller et al. 2006) as are calf/cow estimates, also generated from harvest data (Fig. 2). However, changes in K itself may be occurring due to a warming climate with studies indicating a decline in the Bering Sea (Grebmeier et al. 2006) and an increase in the Chukchi Sea (Sirenko and Gagaev 2007).

## Limiting Factors

### Hunting

Pacific walrus have been hunted for subsistence for millennia, which continues today, commercially since the 17th century (Fay 1982) until 1990, but recreational harvests were minimal. Estimates of harvest levels (Table 1) were originally derived from records of commercial operations and some professional judgment, but since 1960, total harvest removals have been monitored in both the Russian Federation and United States (USFWS, unpubl. data). The history of harvests is one of large fluctuations until a collapse in the early 1900s, and a rebound followed by a consistent and sustainable level (Scammon 1874; Fay 1957; Bockstoce and Botkin 1982; Table 1).

**Figure 3:**
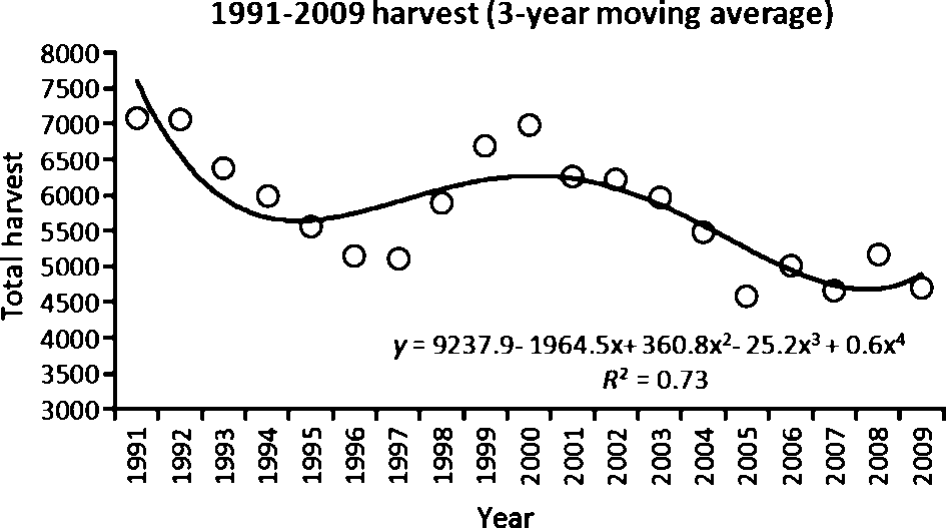
Total annual United States and Russian Federation harvest (3-year moving average) of Pacific walrus from 1990 to 2010, which includes a struck and lost adjustment of 42%. Trend line is from an ordinary least squares regression, presented above the line.

In the 1960s, the State of Alaska and the Union of Soviet Socialist Republics implemented harvest restrictions (Fay et al. 1989; Garlich-Miller et al. 2006) resulting in the population recovering to pre-exploitation levels (Fay et al. 1989). The Marine Mammal Protection Act of 1972 placed a moratorium on the commercial and sport harvest of walrus in United States waters, but exempted subsistence harvests by Alaska Natives. Nonetheless, between 1976 and 1979, the State of Alaska managed the walrus harvest based on a federally imposed quota of 3000/year. Relinquishment of management authority by the State of Alaska to the USFWS in 1979 removed the harvest quota, resulting in increased harvest rates in subsequent years (Table 1). The increased harvest of the 1980s included an increase in the proportion of females harvested, which likely reduced the population (Fay et al. 1997). During this period, commercial harvests by the Russians were also occurring which lasted until 1990. Since 1991, removal levels range-wide were between 3830 and 8518/year, have declined by an average of 2% (SE = 8)/year, but have recently leveled-off (Fig. 3).

### Other anthropogenic factors

Other than hunting, activities of humans have had relatively little direct effect on the Pacific walrus population (Jay et al. 2011). Commercial fisheries may impact walrus through direct mortalities, displacement, bycatch of prey, and modification in prey habitats, but fishing has largely been confined to the southern periphery of the walrus range. Oil and gas exploration can have similar impacts with the addition of noise from seismic surveys, but has occurred in relatively limited areas. Shipping has been limited to supplying coastal villages and constrained by sea ice, but shipping also increases underwater noise and could result in direct mortalities and displacement. Some contaminants occur in walruses (Robards et al. 2009) but have not impacted the population or been a threat to subsistence users.

### Natural factors

Generally, hunting has kept the walrus population below K. However, in the 1980s, harvest restrictions appeared to have been effective, the population increased, productivity declined (Garlich-Miller et al. 2006; Fig. 1), and managers and researchers felt the population had reached or exceeded K and density-dependent mechanisms were limiting population growth (Fay et al. 1989, 1997).

Other natural factors have not been known to limit population growth (Fay 1982; Jay et al. 2011). Little information is available on prey population dynamics, and there has never been a reported die-off of walrus due to starvation, although a few malnourished animals are regularly observed (Fay 1982; Eskimo Walrus Commission [EWC] 2003; EWC, unpubl. data). Predation by polar bears and killer whales does not appear to have ever been great enough to be the primary cause of a population decline, or prevent a population increase following a reduction in human harvests (Fay 1982). No disease epidemic or pandemic has ever been reported in Pacific walrus, but recent observations have documented a low prevalence (≍6% of animals surveyed) of an unknown, possible new disease with symptoms of ulcerative dermatitis and lethargy (Garlich-Miller et al. 2011). The above, and the relatively strong association between harvest levels and population size since the 1970s (*r*_*s*_ = 0.71; Table 1), suggests that other factors affecting the population have had minor effects.

## Climate-Induced Change and Walrus Response

### Sea-ice habitats

**Figure 4:**
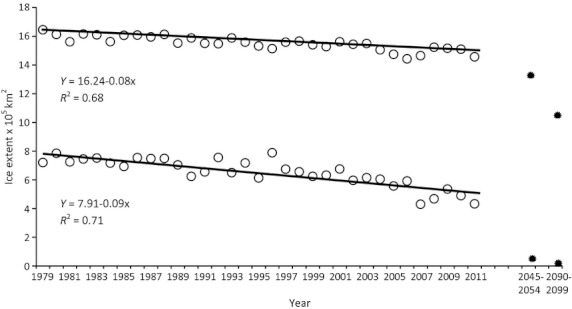
Arctic sea-ice extent (km^2^) for March (annual maximum, upper series) and September (annual minimum, lower series) from 1979 to 2011 estimated from satellite data (open symbols) and median model projections for the 2045–2054 and 2090–2099 decades (closed symbols) from Douglas (2010). Trend line for 1979–2011 is from an ordinary least squares regression, presented below each line; source: National Snow and Ice Data Center, University of Colorado, Boulder, CO.

**Figure 5:**
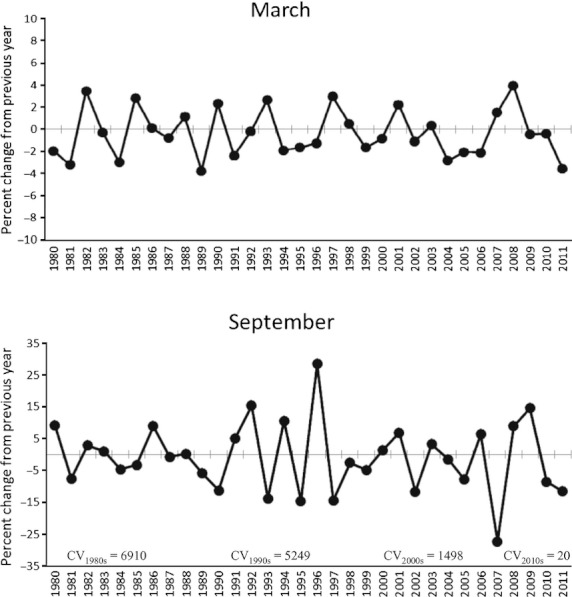
Percent change in sea-ice extent (km^2^) from the previous year for March (annual maximum) and September (annual minimum) from 1979 to 2011 estimated from satellite data; source: National Snow and Ice Data Center, University of Colorado, Boulder, CO. Note differences in *y*-axis scale. CV_i_ is the coefficient of variation (standard deviation [SD]/mean) for that decade.

The driving ecological factor that has changed over the last several decades has been declines in the extent of sea-ice in both winter and summer (Fig. 4). Arctic sea-ice extent (km^2^), derived from passive microwave satellite data, reaches a minimum in September which has been declining over the past 30 years by about 9%/year, with the record low set in 2007 and comparable lows occurring again in 2008, 2010, and 2011 (Fig. 4). The maximum sea-ice extent occurs in March each year and has exhibited a decline of about 0.5%/year, with record lows occurring in 2006, followed by 2011, then 2007 (Fig. 3). However, the percent change in sea-ice extent between consecutive years illustrates how variable those trends are (Fig. 5). For the March record, the mean (standard error [SE]) percent annual change was –0.4(0.4), ranging from –3.8% to 4.0%. The mean annual percent change for September is –1.0(1.9), but ranged from –27.4% to 28.5%. The changes in sea-ice dynamics in the Bering Sea are far less extensive and less variable than those in the Chukchi Sea and both mirror trends for the entire Arctic.

Changes in Bearing Sea-ice dynamics may have impacted Pacific walrus (Jay et al. 2011). Grebmeier et al. (2006) reported a decline in sea-ice cover, benthic macrofaunal mass and sediment oxygen uptake, and an increase in air and water temperature in a relatively small area southwest of St. Lawrence Island. They suggested that a shift from an Arctic to a sub-Arctic ecosystem should be expected for the region and will alter conditions for walrus. However, recent data suggest such a shift has yet to occur as the bottom cold water pool has persisted (Sigler et al. 2011; Stabeno et al. 2012). There are some trends in walrus distribution that could be related to such an ecosystem shift, but also to other causes (Bluhm et al. 2011). For example, many of the traditional Bering Sea summer haulouts are no longer used or contain fewer animals (Metcalf and Robards 2008; Sell and Weiss 2010; Winfree 2010). However, more males joining the spring migration (EWC 2003; Oozeva et al. 2004) and the formation of new haulouts are most likely responsible for that trend. In addition, a change in population size affecting haulout attendance (Fay 1982) cannot be ruled out.

The greatest changes in sea ice within the range of the Pacific walrus have occurred during the melt season in the Chukchi Sea (Jay et al. 2011). September sea-ice extent has declined, with a subsequent ice-free period of two or more months (Table 2). However, more important than ice extent, per se, is the location of the ice edge in relation to the northern edge of the continental shelf, and the persistence of remnant ice over the shelf. When the ice edge moves north of the continental shelf, walrus abandon the ice and within a week or two move to coastal areas to haulout and stage until the southward migration (Jay et al. 2011). If remnant ice persists, as in 2008, walruses will not come to shore. The number of walruses occupying coastal haulouts along the Chukchi Sea has increased by an order of magnitude since 2000 and some haulouts are occupied by many tens of thousands of animals. In October 2010, scientists counted 120,000 walrus hauled out at Cape Serdse Kamen in Chukotka, Russia (Kochnev, unpubl. data 2012; USFWS, unpubl. data 2012).

**Table 2:** Observed and predicted changes in the environment of the Bering and Chukchi Seas and in Pacific walrus behavior and ecology. Predictions are given for most likely- (MLC), worst- (WC), and best-case (BC) scenarios

		Predicted	
		
Area and factors	Observed^1^	2045–2054	2090–2099
Bering Sea			
Sea-ice extent (March, 10^5^ km^2^)^2^	15.1	12.5–13.7	10.0–10.2
Ice-free months/year^1^	5.5	6.4–6.9	8.4–8.9
Ice edge December–February	57–64°N latitude	58–67°N latitude	61–69°N latitude
Prey abundance/composition, walrus response	Possible decline, no change in diet	BC: stable-moderate decline, prey switching WC: large decline- extirpation, no prey switching	BC: stable-moderate decline, prey switching WC: extirpation, no prey switching
Haulout occupancy and size	Variable, declining	MLC: no change BC: stabilizes WC: abandoned	BC: no change WC: abandoned
Haulout mortalities	Variable, no affect on recruitment	MLC: no change BC: no change WC: no change	BC: no change WC: no change
Subsistence harvest trends, sustainability	Stable-declining, sustainable	MLC: stable, sustainable? BC: no change WC: increases, unsustainable	BC: regulated, sustainable WC: stable-increases, unsustainable
Resource development	Increasing, mitigated	MLC: no change WC: larger increase, mitigation ineffective BC: stabilizes-declines	BC: stabilizes-declines WC: larger increase, mitigation ineffective
Chukchi Sea			
Sea-ice extent (September, 10^5^ km^2^)^1^	5.6	0.2–0.4	0
Ice-free months/year^1^	0.9	2.1–2.2	4.1–4.3
Ice edge in September	72–75°N latitude over the continental shelf	76–80°N latitude over the Arctic Basin	>80°N latitude over the Arctic Basin
Prey abundance/composition, walrus diets	High mass and diversity, bivalves, snails, worms	BC: change in species, prey switching WC: decline-extirpation, no prey switching	BC: change in species, prey switching WC: extirpation, no prey switching
Haulout occupancy and size	Increasing occurrence, duration 3 months, 1–100 × 10^3^ animals	MLC: regular occurrence, duration increases, 1–100 × 10^3^ animals WC: same as MLC BC: no change	WC: regular, duration increases, 1–10 × 10^3^ animals BC: no change
Haulout mortalities	Variable, 100s-1000s, short-term effect on recruitment	MLC: no change BC: declines, no effect on recruitment WC: less variable, increases, affects recruitment	BC: no change WC: less variable, increases, affects recruitment
Subsistence harvest	Stable-declining, sustainable	MLC: stable, unsustainable WC: increases, unsustainable BC: no change	BC: stable-increases, sustainable WC: increases, unsustainable
Resource development	Increasing but mitigated	MLC: no change BC: stabilizes WC: widespread increase, no mitigation	BC: no change WC: widespread increase, no mitigation
Range-wide			
Population size	>129,000	MLC: declines to 10^3^–10^4^ + WC: declines to 10^3^–10^4^ + BC: no change	BC: declines 10^3^–10^4^ + WC: declines to ≤10^2^
Distribution	Winter and breed in northern Bering Sea, males summer in Bering Sea, females summer in Chukchi Sea	MLC: northward shift BC: no change WC: winter and breed further north, summer no change	BC: winter and breed further north WC: winter and summer in Chukchi Sea or move to Russian/Canadian Arctic
Migrations	Spring earlier, fall later, routes variable	MLC: slight change BC: no change WC: increased change	BC: increased change WC: no migration, stay in Russian/Canadian arctic
Female productivity (calves/cow)	0.4–0.7	MLC: no change BC: no change WC: decline (0.2–0.4)	BC: no change WC: decline (0.2–0.4)
Pollution/contaminants	Low exposure, low tissue concentrations	MLC: increased exposure, low tissue concentrations WC: increased exposure, high tissue concentrations BC: no change	WC: increased exposure, high tissue concentrations BC: increased exposure, low tissue concentrations

1 Generally, last two to three decades depending on subject.

2 Based on Douglas (2010), observed = 1979–2008, ranges = median decadal projections of SD1 and SD2 model subsets for A1B and A2 emissions scenarios for ice extent, and the A1B scenario for ice-free months.

In addition to the increased use of coastal haulouts, changes in sea-ice dynamics have also affected the migration patterns of walrus (EWC 2003; Oozeva et al. 2004; EWC unpubl. data). Many hunters in Alaska indicate that the spring migration occurs about a month earlier than in past decades, is more rapid, and routes may have changed. The fall migration also begins a month or so later.

There are two concentrations of Pacific walrus during the summer in the Chukchi Sea when ice is present over the continental shelf (Fay 1982), one immediately west of the northwest coast of Alaska in the Hanna Shoal area, and another to the west in the vicinity of Wrangel Island, Russian Federation. As the ice moves off the continental shelf, the group near Wrangel Island moves to the Island (Fay 1982) or more recently, mainland Chukotka and the group off the coast of Alaska moves southeast. In recent low ice years, walruses have hauled out at several locations along the Alaska coast in August and September. In 2010 and 2011, ≍20,000–30,000 animals have hauled out near the village of Point Lay, Alaska, for the first time. The Alaska animals subsequently move south along the coast in advance of the developing ice pack until reaching Cape Lisburne, at which point they move to the Russian mainland, landing near Cape Serdtse-Kamen (Fischbach 2012; http://alaska.usgs.gov/science/biology/walrus/2011animation_Norseman.html).

There are two primary stressors associated with the recent trend of sea-ice loss from shallow continental shelf areas of the Chukchi Sea; (1) animals that stay with the sea ice as it moves over the Arctic basin cannot feed and become emaciated and weak before eventually abandoning the ice for the coast, and (2) mortalities due to crushing of young animals at coastal haulouts can number in the hundreds to thousands (Fischbach et al. 2009; Jay et al. 2011). However, both stressors seem to have moderated since the extreme events of 2007. In 2008, there was enough remnant ice to allow walrus to remain offshore, and mitigation efforts at coastal haulouts in both the Russian Federation and United States have reduced human-caused disturbance and mortalities of young animals (USFWS 2012).

### Hunting

In Alaska, subsistence hunters have noted that changes in sea ice during traditional hunting periods have affected the movements and distribution of walrus (EWC 2003; Oozeva et al. 2004). In addition, increases in the number of days with high winds and rough seas have reduced opportunities to hunt (EWC 2003). Unfavorable weather conditions and walrus migrating further from villages may be contributing to the overall small decline in harvests (Hovelsrud et al. 2008). Another factor that may be important was the adoption of limits on the number of walruses that could be harvested during each hunting trip by the Native Villages of Gambell and Savoonga, Alaska (responsible for 80–90% of the walrus harvest in Alaska). Alternatively, the walrus population may have declined in response to observed ecological conditions and hunting pressure, and reduced abundance is also limiting hunting success (Garlich-Miller et al. 2006). However, there is some evidence that hunters are adapting to changing conditions (Fig. 2) by extending the traditional hunting season, that is, by beginning earlier and hunting at new fall haulouts in Alaska. Although harvest levels are lower than historic highs, the lack of information on walrus population size and demographics preclude an accurate assessment of the sustainability of recent harvest levels.

### Ocean warming and acidification

The surface waters of the Arctic Ocean and surrounding seas, including the Bering and Chukchi Seas, have warmed (Steele and Boyd 1998; Zhang et al. 1998; Overland and Stabeno 2004; Stabeno et al. 2007; Mueter et al. 2009; Steele et al. 2008). Grebmeier et al. (2006) documented a 1.5°C increase in the subsurface cold water pool near St. Lawrence Island in the Bering Sea, but that did not persist (Stabeno et al. 2012). Due to their tolerance of considerable variations in temperature, direct effects to walrus have not been observed nor anticipated with warmer ocean temperatures. Nevertheless, changes in the thermal dynamics of ocean conditions may affect walrus prey. Benthic productivity on the northern Bering Sea shelf has decreased over the last two decades, coincident with a reduction of northward flow of the Anadyr current through the Bering Strait (Grebmeier et al. 2006).

The ocean is a sink for atmospheric carbon, absorbing about one-third of the atmospheric CO_2_ (Feely et al. 2004; Canadell et al. 2007). When CO_2_ is absorbed by seawater, chemical reactions occur that reduce seawater pH and the concentration of carbonate ions, in a process known as “ocean acidification” (OA). The absorption of carbon dioxide by seawater reduces the concentration of aragonite, which is important in the Arctic because clams, mussels, snails, crustaceans, and some plankton use aragonite in their shells and exoskeletons (Fritz 2001; Fabry et al. 2008; Steinacher et al. 2009). The effects of ocean acidification on walrus may be through changes in their prey base, or indirectly through changes in the food chain on which their prey depend; however, such changes have not been documented. Ridgwell et al. (2009) suggested that the effects of OA may not be observable for another 30 years, at least in zooplankton communities, due to wide variation in zooplankton tolerance to OA as well as ocean carbon cycles and integrating patterns. In addition, Cai et al. (2010) suggest that CO_2_ uptake by the Arctic Ocean may have reached an asymptote as air and surface water concentrations equilibrate due to a shallow mixed-layer depth, strong surface water stratification, surface warming, and low biological CO_2_ fixation.

To date, there is no direct evidence that changes due to ocean warming or acidification have resulted in alterations to walrus behavior, distribution, or population dynamics. However, these factors may be contributing to the general northward shift in distribution and declining occupancy of Bering Sea coastal haulouts.

### Commercial fishing

Commercial fisheries can impact Pacific walruses through direct interactions such as ship strikes and entanglements and indirectly through competition for prey resources and destruction of benthic prey habitat. Incidental mortality during 2006–2010 was observed during only one fishery, the Bering Sea/Aleutian Island flatfish trawl with a median number of observed mortalities of two walruses/year (range 0–3; USFWS 2012).

Bottom trawl fisheries also have the potential to indirectly affect walruses through destruction or modification in benthic prey and their habitat. McConnaughey et al. (2000, 2005) reported some large differences in walrus prey in the Bering Sea between heavily trawled and untrawled areas. However, those studies were based on trawl gear that is no longer in use in those areas (National Oceanic and Atmospheric Administration [NOAA] 2010).

Commercial fisheries currently occur only in Kotzebue Sound in the Chukchi Sea, but fishermen are interested in following stocks north if fish distribution changes. Even then, it appears that those activities would be adequately researched and well regulated (USFWS 2011).

### Oil and gas development

Oil and gas exploration activities in the Chukchi Sea in both the United States and Russian Federation have increased over the last decade (USFWS 2011). To date, exploration has been limited to seismic surveys and exploratory drilling. Mitigation measures (USFWS 2008) and associated monitoring programs suggest that impacts to walruses have been limited (Reiser et al. 2010).

### International commercial shipping

Shipping traffic within the range of Pacific walruses has been fairly limited in the past primarily due to persistent pack ice in the region. As ice conditions moderate shipping levels are increasing. For example, shipping activity (number of transits) through the Bering Strait increased by 7–423% from 2009 to 2010, with the largest increase occurring west of the international dateline (WWF, unpubl. data). The U.S. Coast Guard is currently assessing potential commercial ship routing through the Bering Strait region (United States Coast Guard [USCG] 2010) which will be considered in the development of regulations to govern shipping in United States waters. In addition, the Russian Federation complies with a number of voluntary guidelines, but is not a party to other agreements (Molenaar 2009).

## Methods

Forecasts of sea-ice dynamics by Douglas (2010) for the Bering and Chukchi Seas drive predictions of future ecosystem characteristics. In addition, paleoecological research, walrus life history characteristics, and recent observations (USFWS 2011) are used to predict how Pacific walrus will respond to changing sea-ice ecosystems. Douglas' (2010) forecasts indicate more years with less ice cover and an extended ice-free season in both the Bering and Chukchi Sea, with the greatest changes occurring in the Chukchi Sea. In addition, the position of the edge of the pack ice will be north of the continental shelf from August through October–November (Table 2). These changes in sea-ice dynamics influence nearly every aspect of not only Pacific walrus ecology but also human activities that potentially affect walrus. In addition, these factors interact in complex ways and may have cumulative effects. The magnitude and interactions of various future stressors are difficult to predict, likely highly variable (Brock and Carpenter 2010), and potentially novel (Williams and Jackson 2007).

Due to a lack of data, the complexities of the Pacific walrus–climate warming interactions, and many unknowns, Jay et al. (2011) and MacCracken et al. (2012) developed Bayesian belief network models (BBN) to assess the cumulative effects of a variety of stressors on the Pacific walrus, covering past and present conditions and making forecasts to the end of the 21st century. Sensitivity analyses of both models identified habitat change (and associated stressors) and subsistence harvest as the most important stressors influencing model outcomes and the most important stressor associated with habitat change was mortalities of young animals on coastal haulouts.

MacCracken et al. (2012) modeled conditions at mid-century (2045–2054) and late-century (2090–2099) decades with three scenarios: most likely-case (S_mc_), best-case (S_bc_), and worst-case (S_wc_). The most likely-case represents scenarios judged to be the most plausible; the best-case and worst-case scenarios are less likely to occur; however, one or the other often aligned closely with the most likely-case (Table 2). Due to many uncertainties, a plausible most likely-case scenario for the late-century decade was not developed. Each scenario represents a unique combination of sea-ice dynamics and the intensity of associated stressors. The results of the scenarios were then incorporated into four comprehensive working hypotheses (Chamberlin 1890; reprinted in *Science* [1965]). In short, Chamberlin advocated the development and consideration of all plausible and rational hypotheses that potentially explain new phenomena to counteract biases introduced through a singular focus on a “ruling hypothesis” or a “single working hypothesis”. Chamberlin's (1890) method of multiple working hypotheses is one of the philosophical underpinnings of modern model selection approaches to data analyses (Burnham and Anderson 2002; Elliott and Brook 2007), and Elliott and Brook (2007) noted that the method of multiple working hypotheses, in contrast to the classic analytical method (Platt 1964), allows for the existence of more than a single explanatory hypothesis – a distinct possibility given the complexity of Pacific walrus–climate warming interactions.

The method of multiple working hypotheses has relevance to management and research programs as it can help identify and prioritize climate change responses (Gray 2011). Commonalities in conditions and outcomes among scenarios point to actions that can be taken immediately, regardless of how the future develops; as the future begins to align with any one hypothesis, managers can predict potential outcomes and make adjustments as necessary and researchers can identify the most crucial management and policy relevant projects. Because conditions vary substantially between the Bering and Chukchi Sea in terms of environmental changes, subsistence hunting, resource development, and walrus occupancy and behavior, I discuss each area separately and then consider circumstances that apply range-wide.

## Results

### Bering Sea

In the Bering Sea, maximum ice extent (cover) is predicted to decline (from a 1979–2008 baseline) about 4% by mid-century and 20% by late-century, resulting in a 1- and 3-month increase in the number of ice-free months, respectively. The position of the ice edge does not move north appreciably until late-century (Table 2). The most important aspect of these ice changes is likely on walrus breeding behavior and the location of breeding aggregations (USFWS 2011). Walrus begin to gather at traditional breeding sites in the Bering Sea in December and remain in those areas until February. Projections for those months put the ice edge about 1–3° latitude north of 1979–2008 observations at mid-century (Table 2). By late-century, the ice edge will be 4–5° north of the baseline location. S_mc_: because walruses will continue to breed where females are aggregated on the ice, a northward shift in breeding sites will occur. The effects of a northward shift in breeding are presumably minor. No alternatives to this scenario were deemed plausible.

There are indications of a decline in benthic mass in the Bering Sea (Grebmeier et al. 2006), but it is not known if that decline includes walrus food items. Observations of large numbers of malnourished animals among walrus summering in the Bering Sea have not been made. Potential changes in walrus prey due to ocean warming and acidification (OA) have not been documented (Jay et al. 2011). If temperature changes and OA in the Bering Sea affect walrus prey, there will most likely be changes in species composition and abundance due to variation in susceptibility of different species at different life stages, that is, there will likely be ecological winners and losers (Hendricks et al. 2010; Kroeker et al. 2010; Hale et al. 2010; Ocean Acidification Task Force [OATF] 2011). In addition, benthic species from warmer waters may move north (Grebmeier et al. 2006; Sirenko and Gagaev 2007; Hansen et al. 2011). Walrus diet diversity (Sheffield and Grebmeier 2009) suggests some flexibility in prey selection and they may be able to adapt (to an unknown extent) to a changing prey base. S_bc_: these processes may result in little change or a small-moderate overall decline in benthic invertebrates that will be suitable as walrus prey. S_wc_: alternatively, changing ocean conditions could lead to the extirpation of the majority of preferred prey and species that may fill those vacant niches may not be suitable as walrus prey. Due to a lack of detailed and current information on walrus prey, a most likely-case scenario could not be developed for this stressor.

Occupancy and size of Bering Sea coastal haulouts varies daily and annually (Sell and Weiss 2010; Winfree 2010) and that pattern of variation is not expected to change (Table 2). Bering Sea coastal haulouts are dominated by adult males. Some coastal haulouts are no longer used (Jay and Hills 2005) and numbers of animals at other haulouts have steadily declined by 2–3% each year over the last three decades (Sell and Weiss 2010; Winfree 2010; Zdor et al. 2010); however, new haulouts have formed during that same period. Declines in haulout attendance could be due to a decline in prey, a decline in the population, the movement of males to the Chukchi Sea in the summer (EWC 2003), the use of new haulouts, or all four. In addition, the possible decline in prey could be due to over-exploitation by walruses, ecosystem changes, spatial shifts in distribution (Bluhm et al. 2011), or most likely a combination of these factors. S_mc_: the decreased use of some Bering Sea coastal haulouts is expected to continue with a concomitant increase at another. S_bc_: conditions stabilize in the future around a lower level of haulout attendance due to density-dependent feedback mechanisms. S_wc:_ Bering Sea coastal haulouts are abandoned for sites further north (Table 2).

Mortalities at Bering Sea coastal haulouts occur for a variety of reasons (Fay 1982; Winfree 2010; USFWS 2011) and most are not related to habitat changes. S_mc_: mortalities at Bering Sea haulouts are expected to continue but not at levels that would contribute to a sustained population decline; plausible unique best-case and worst-case scenarios were not developed (Table 2).

Eighty to ninety percent of walruses harvested in the United States are taken near St. Lawrence Island (USFWS 2011) in the northern Bering Sea. Subsistence harvest levels have been declining by about 2%/year since 1991, but have recently leveled-off (Fig. 3). I expect this trend to continue, but others have predicted more substantial declines in harvest levels of walruses and other marine mammals, primarily due to habitat changes reducing hunter opportunities and success (Hovelsrud et al. 2008). S_mc_: harvest levels remain relatively stable and the sustainability of the harvest remains unknown. S_wc_: harvest levels increase or remain stable, and the harvest becomes unsustainable because the walrus population declines substantially and hunters do not limit their take. S_bc_: hunters acknowledge a population decline and take actions to reduce harvest impacts or legal mechanisms to set quotas in the United States come into force, largely through a change in status from a candidate for listing under the ESA (USFWS 2011) to threatened or endangered.

Commercial activities in the Bering Sea are largely associated with a variety of fisheries, the shipping of goods to coastal communities, logistical support of development activities such as oil and gas exploration, and recreation/tourism. These activities are increasing and for the most part, potential impacts are minimal and mitigated through regulations (USFWS 2011). Oil and gas leases that could have impacted Bristol Bay were withdrawn from consideration in 2010. Increases in international commercial shipping activity, passenger vessels, and tourist cruises are likely to continue (Hovelsrud et al. 2008; Arctic Council 2009; Brigham 2011) and may outpace regulatory programs (United States Coast Guard [USGC] 2011). S_mc_: resource development in the Bering Sea will continue to increase and current activities will be adequately mitigated. However, economic conditions could change resulting in; S_bc_: a stable or declining level of activities, or S_wc_: a widespread increase and a relaxation of regulations and monitoring requirements (Table 2).

### Chukchi Sea

Sea-ice extent in September in the Chukchi Sea has declined and is expected to be nonexistent by late-century (Douglas 2010) or sooner (Wang and Overland 2009). This will result in the average number of ice-free months/year increasing from about one currently to four or five by late-century. More importantly for walruses, the ice edge will move further north, off the continental shelf, and over the Arctic Basin sooner in the summer (Douglas 2010), resulting in walrus abandoning the ice earlier and using coastal haulouts for longer periods (Table 2). Other model projections have estimated that by mid-century, the entire Arctic will be ice-free in August–September (Stroeve et al. 2007; Wang and Overland 2009), which suggests that conditions predicted for late-century in the Chukchi Sea will be reached sooner.

Prey abundance in the Chukchi Sea is relatively high, but not uniformly distributed (Grebmeier et al. 1989). Sirenko and Gagaev (2007) reported an increase in the abundance of chalky macoma (*Macoma clacera*) in the Chukchi Sea as well as the recent northward expansion of crabs and a bivalve from the North Pacific. However, as for the Bering Sea, the effects of temperature increases and OA on specific walrus prey species have not been studied. If OA and temperature changes in the Chukchi Sea affect walrus prey, there will most likely be changes in species composition and abundance as postulated for the Bering Sea; however, the data of Sirenko and Gagaev (2007) are not in agreement with that of Grebmeier et al. (2006). In addition, walrus response will likely follow the same two paths; S_bc_: adaptation through prey switching and increased use of increasing bivalves, or S_wc_: walrus will not be able to adapt. In the latter scenario, K will decline, female condition and productivity will decline, and the population will decline.

The trend of increasingly large numbers of animals concentrating in a relatively few areas of the Chukotka coast for longer periods each fall is expected to continue. This situation could result in the depletion of prey in those areas, which may limit the walrus population because other areas that could be used by walrus are unlikely to provide similar levels of prey (Grebmeier et al. 1989). Haulouts on the Alaskan coast will likely become more frequent and predictable in the short term, but benthic mass estimates (Grebmeier et al. 1989) and movement patterns of walrus once they reach the coast (Fischbach 2012) suggest that in the long term, use of the Alaskan coast may not persist. S_mc_: walrus recover from a period of reduced prey intake when sea-ice returns in early-winter and once again provides access to more foraging areas. S_bc:_ walrus prey remains abundant enough to support the current or a slightly smaller population. S_wc_: the walrus population is limited by prey that can be obtained from coastal haulouts and the population declines substantially (Table 2).

Mortalities of young animals at coastal haulouts vary from year to year, but are likely to always occur at some baseline level. S_mc_: current and developing management programs at coastal haulouts in Chukotka and Alaska reduce human-caused mortality events and continue to be effective. However, despite those efforts, large mortality events may still occur, the frequency of which will determine impacts on the female breeding cohort in subsequent years. Ideally, these events occur at annual intervals that result in small age-class gaps in future breeding cohorts. S_wc_: large numbers of mortalities at haulouts occur annually, depressing recruitment, and resulting in a large population reduction. S_bc_: haulout mortalities decline and stabilize at background levels (Table 2).

Subsistence demand for walruses by Chukchi Sea communities in Alaska is far less than that of the northern Bering Sea/Bering Strait communities (Hill 2011). However, fall coastal haulouts provide additional hunting opportunities to Chukchi coastal residents. To date, reported hunting of walrus on Chukchi coast haulouts in Alaska has been limited to a few animals each year and that situation is expected to continue. In addition, the Chukotka take is governed by a quota set each year by Russian authorities, which has progressively gotten more restrictive (USFWS 2011). S_mc_: take by Chukchi coastal residents remains stable or increases slightly. S_wc_: the overall subsistence harvest becomes unsustainable, and harvests in the Chukchi Sea increase or remain at current levels contributing to the problem. S_bc_: hunters in Chukchi Sea communities adjust harvest levels according to population trends (Table 2).

Development activities in the Chukchi Sea are increasing, and the loss of sea ice in the summer and fall is facilitating those pursuits and creating opportunities for additional undertakings, primarily international shipping (USFWS 2011). We expect that trend to continue, but the overall footprint of these activities is small and regulations governing oil and gas exploration and development, at least in United States waters, mitigate impacts to walruses (USFWS 2008, 2011). Although statistically infrequent, an accident leading to a large release of oil or other contaminants into the Chukchi Sea could have severe consequences to the Pacific walrus population depending on amount, timing, containment success, and walrus distribution.

International shipping through the Chukchi Sea is increasing and commercial fishing activities are limited (USFWS 2011; Wilson 2006). Commercial fishing in the United States is most likely to expand relatively slowly, if at all, and be adequately regulated. Shipping is becoming more frequent and is expected to increase with rates and patterns of increase following one or more scenarios (Arctic Council 2009), which dictate the effects of increased shipping on walruses and other entities. However, arctic shipping expansion appears to be ahead of regulation development and that pattern is likely to persist in the short term.

S_mc_: commercial activities continue to increase in the Chukchi Sea; most activities are localized and adequately regulated. Shipping increases and the U. S. Coast Guard defines shipping lanes and seasons of use that limit/mitigate potential impacts. An accident that releases large amounts of oil or other contaminants does not occur. S_wc_: activities increase and become widespread, regulations are not developed, relaxed or rescinded, and a large oil spill occurs that impacts thousands of walruses and thousands of km^2^ of habitat. S_bc_: exploitable oil and gas deposits are not discovered, development declines, activities remain localized, and a large demand for international shipping does not materialize.

### Range-wide

Range-wide considerations include population size and distribution, migration behaviors, female productivity in terms of calf/cow estimates, disease and predation, pollution and contaminants, and international commercial shipping (Table 2). S_mc_: habitat changes and associated stressors result in a population decline and a continued northward shift in distribution, spring migrations continue to occur earlier and faster, female productivity remains relatively high, disease and predation continue to have a minor role in walrus population dynamics, and pollution and contaminants increase slightly. S_wc_: the population declines rapidly and substantially, the arctic becomes ice free in summer by mid-century and walruses migrate to sea-ice refugia in the Siberian-Laptev Seas and the Canadian Arctic, female productivity declines due to a reduction in suitable prey and an increase in pollution and contaminants. S_bc_: sea-ice changes are not as rapid or large as predicted, the walrus population stabilizes, the northward shift in distribution stabilizes, walrus prey remains relatively abundant, northward expanding benthic species are suitable prey, female productivity remains high, and no increase in pollution or contaminants occurs.

### Working hypotheses

The following working hypotheses (WH_i_) cover the range of plausible future conditions based on the cumulative effects of the stressors and result in divergent outcomes. Other potential scenarios considered incorporated small and subtle changes in the intensity of one or a few stressors, and they would fall within the bounds of these hypotheses. Because of the intricacies of each hypothesis, I did not attempt to determine a most likely-case, but it should be apparent which would represent best-case and worst-case scenarios.

WH_1_: Pacific walrus continue to occupy both the northern Bering and Chukchi Seas, but use of the southern Bering Sea continues to decline. Summer-fall Chukchi coastal haulout use increases in duration, mortalities at haulouts stabilize at a sustainable level but large events occur periodically (e.g., every fourth year), foraging from fall haulouts limits the population, subsistence harvest remains stable, resource developments increase but are mitigated, and no large industrial accidents occur. Under this hypothesis, the population will decline to a level dictated by prey resources that can be accessed from fall coastal haulouts and most likely fluctuate at that level. The information needed to reliably predict the rate or magnitude of the population decline is not available. Three areas of major uncertainty in this scenario are the effects of OA and temperature on walrus prey, harvest sustainability, and the effects of an industrial accident.

WH_2_: sea-ice predictions for 2099 occur by mid-century, the northward shift in walrus distribution increases, fall coastal haulout use increases in frequency and duration, frequent large mortality events occur at haulouts, foraging from summer-fall haulouts limits the population and OA and temperature increases further reduce walrus prey, subsistence harvest becomes unsustainable due to a large and rapid population decline, resource developments increase, and impacts are inadequately mitigated. Under this hypothesis, I expect the population to decline to a point where the probability of extinction increases substantially.

WH_3_: under either of the above hypotheses, over the long-term walruses eventually migrate from the Bering and Chukchi Seas to sea-ice refugia in the eastern Siberian-Laptev Seas and the Canadian Arctic where the population either stabilizes at the carrying capacity of those areas or declines to lower numbers.

WH_4_: economic forces, technological advances, and policy changes result in reduced greenhouse gas emissions, sea-ice dynamics moderate and ice recovers over the next few decades (Amstrup et al. 2010; Serreze 2011), fall haulout use and associated mortalities decline, the population is limited primarily by the subsistence harvest, and resource development increases but is seasonally restrained by sea ice and adequately regulated. Under this hypothesis, I expect the population to decline until sea ice recovers to near previous patterns and then to increase or fluctuate at a reduced abundance due to subsistence harvest.

## Discussion

The primary extrinsic factor currently affecting Pacific walruses is the reduction of sea ice in the summer/fall over the continental shelf of the Chukchi Sea. The primary intrinsic factor is large coastal haulout mortalities that are linked to disturbance events. Extrinsic factors often manifest change that is predictable, spatially uniform, and linear, whereas intrinsic change is often nonlinear and spatially and temporally variable (Williams et al. 2011). These generalities fit the current state of climate warming–Pacific walrus interactions. We have observed a steady, but annually variable, decline in sea-ice extent and northward advance of the ice edge each summer, which is predicted to continue in a linear fashion (Douglas 2010). However, mortalities at haulouts have been highly variable both in time and space, particularly along the Alaskan coast (USFWS, unpubl. data). Change due to extrinsic factors invokes questions of adaptability and resilience, while intrinsic factors can reveal the mechanisms that lead to regime shifts when thresholds or tipping points are crossed (Williams et al. 2011). Brock and Carpenter (2010) examined changes in variance structure as systems neared thresholds and suggested that monitoring of variance components could act as an early warning system of impending regime shifts. The greater variation in annual percent change in sea-ice extent in September during the 1980s and 1990s and subsequent reduction in the 2000s (Fig. 5) illustrate this point.

Species responses to rapid and large-scale habitat changes take three paths: in situ adaptation, dispersal, and extinction which can be affected by behavioral, physiological, and anatomical plasticity (Hof et al. 2011). A regime shift from females with young summering on the ice in the Chukchi Sea to the use of coastal haulouts for several months is well underway. It also appears, based on benthic productivity estimates (Grebmeier et al. 1989; Sirenko and Gagaev 2007), that the Chukotkan coast provides better foraging opportunities, and walrus movements subsequent to ice loss over the shelf (Fischbach 2012) suggest that use of the Alaskan coast may eventually decline in terms of numbers of walruses and duration. Furthermore, as sea-ice loss increases in the future, Pacific walruses may eventually disperse to the northwest (Jay et al. 2011), or northeast (Hill 2011), but for the short term, potential adaptations to conditions in the Chukchi Sea should be the focus of management and research programs.

Pacific walrus can adapt to the loss of sea ice during the summer and fall (Laidre et al. 2008), at least in the short term by hauling out on shore. Use of coastal haulouts in the Bering Sea by males is common and longstanding and large haulouts also formed infrequently along the Chukchi coast in the past (Fay 1982). However, it is clear that sea ice provides females with dependent young superior habitat. Coastal haulout inferiorities manifest primarily as mortalities of young animals due to crowding (background mortalities) and disturbance events (acute, episodic mortalities). Although mortality events at coastal haulouts have been large, apparently these events have not resulted in a large population decline, based on the 2010 fall haulout count in Russia (A. Kochnev, pers. comm. 2012) and the observations of Alaskan hunters (EWC, unpubl. data).

Another major consideration associated with coastal haulout use is the increased energetic expenditure by adult females to acquire the level of prey needed for maintenance, gestation, and lactation (Metcalf and Robards 2008; USFWS 2011; Jay et al. 2011). Evidence of females in poor body condition is inconsistent both spatially and temporally, and more anecdotal than programmatic. Estimates of calf/cow ratios from harvest data suggest that the prey base has been adequate overall and foraging from coastal haulouts has not yet affected population performance. However, as use of coastal haulouts repeats each fall in the same areas, and the ice-free season increases in length, the disadvantages of coastal haulouts will begin to exert more of an influence on walrus population dynamics.

### Management considerations

Facilitating the adaptation of wildlife to climate change is a management priority at international, national, and local levels (Glick et al. 2011). BBN model sensitivity analyses indicated that the most important stressors on the Pacific walrus population, in order of importance, were habitat modification, calf mortalities, summer sea ice, harvest, prey, and greenhouse gas emissions (the latter three were tied; MacCracken et al. 2012). Furthermore, scenario analyses found that mitigation could substantially reduce the intensity of those stressors. That analysis did not include a scenario where greenhouse gas emissions were stabilized or reduced.

Preventing disturbance of walruses while on coastal haulouts is one of the primary actions that can facilitate adaptation to sea-ice changes (Robards et al. 2009) and is applicable under any of the working hypotheses. In most years, it may be possible to predict the timing of Chukchi coast haulout formation based on ice cover as quantified by satellite derived data (National Snow and Ice Data Center 2012). Knowing when to expect a haulout to form would be useful in initiating monitoring programs as well as alerting coastal communities, regulatory agencies, commercial shipping and air carriers, tourism operators, etc. to be vigilant. Once a haulout begins to form, more specific and formal actions can be taken, for example, airspace closures, establishment of buffer zones, hunting management, village-based haulout protection, and monitoring.

Another stressor where mitigation actions are most practical, also applicable under any future scenario, and can facilitate walrus adaptation is the subsistence harvest. Walrus harvest management is relatively straightforward and has been successful in the past (Fay and Bowlby 1994). The exemption of Alaskan Natives from hunting restrictions associated with the Marine Mammal Protection Act currently makes implementing western science-based harvest management practices problematic (Robards et al. 2009; USFWS 2011). However, Pacific walrus are a candidate for listing under the ESA and revising their status to threatened or endangered allows for the development of harvest quotas.

Hunters in the Bering Strait region may be the last to perceive a walrus population decline because animals are concentrated within a relatively narrow corridor as they migrate through the region and changes in weather patterns are also limiting opportunities for hunters to observe population trends. However, hunting ordinances adopted in 2010 by the primary walrus hunting communities in the United States are consistent with traditional practices of self-regulation and making full use of harvested animals (EWC 2003). There is concern among many hunters that these traditions are being lost as elders pass on and younger hunters attempt to integrate traditional values with the economic pressures of western culture (EWC 2003). Walrus are a source of cash income as tusks can be sold and traded among Alaskan Natives and sold to anyone after being substantially altered as handicrafts. This creates an economic incentive to harvest animals solely for tusks, over and above food needs, which increases with inflation and market expansion. Regulations prohibit such activity, but infractions occur, and the vastness of the region complicates enforcement. Programs that facilitate the sale of raw ivory and handicrafts, such as ivory marketing cooperatives, have the potential to maximize the value of walrus ivory and possibly reduce the take of animals solely for tusks, but face many of the challenges of other minority economic cooperatives (Rotan 1995).

Perhaps most important in terms of sustainable harvests and walrus adapting to climate change is limiting the take of breeding females (Robards et al. 2009). Some walrus hunting communities prefer females and young for a variety of reasons (EWC 2003; Robards et al. 2009; Zdor et al. 2010) and reconciling this conflict may not be easy. One measure that could reduce the overall take of females would be a substantial reduction or elimination of females that are mortally wounded and lost. Estimates of the proportion of all animals stuck and lost range from 42% (Fay et al. 1994) to 9% (Kochnev 2010). Kochnev (2010) suggested that the 9% estimate was biased low to an unknown degree and other struck and lost estimates in his report ranged from 10% to 34%. Improvements in weaponry and hunting methods can reduce this factor and actions that would facilitate such improvements should be implemented.

Reliable estimates of population size, which have eluded managers to date (Robards et al. 2009; Speckman et al. 2011), will be needed to insure that the harvest remains sustainable. Methods other than aerial surveys, such as genetic mark-recapture or high-resolution satellite images, may be more informative. The concentration of the majority of the population along the Russian and United States coast in the fall eliminates some of the problems associated with past aerial surveys, but likely introduces new challenges.

If population size cannot be estimated repeatedly and with suitable accuracy, demographic parameters such as survival rates, age and sex composition counts, age at first reproduction, calf/cow ratios, and pregnancy rates may have to suffice (Garlich-Miller et al. 2006; Robards et al. 2009). Fall coastal haulouts also provide a platform for efficient estimation of some of these indices, for example, sex and age composition, but not others, for example, calf/cow ratios. Data collected from harvest monitoring programs (USFWS, unpubl. data; Kochnev 2010) may also provide useful indices of population trend and status. As Robards et al. (2009) noted these indices will be most useful when taken together under a weight of evidence approach.

Walrus summering off the coast of Alaska concentrate in an area of shallow water known as Hanna Shoal (Fischbach 2012). This area is also part of the oil and gas lease sale 193 and is about 50–90 km northeast of the areas that have seen extensive seismic surveys and will eventually be the site of exploratory wells (USFWS 2011). Estimates of benthic mass for Hanna Shoal are high, but lower than for the Chukotka coast (Grebmeier et al. 1989), and the shallow depth presumably influences walrus use of this area. Once the ice recedes beyond the shelf, walrus appear to remain in this area for about 1–2 weeks until they come to shore. Actions that could facilitate the continuation of walrus foraging in this area (e.g., artificial resting platforms, modifications to industrial activities, minimizing benthos disturbance, and enhancing benthic productivity) may need to be undertaken.

Range-wide changes in the Pacific walrus prey base due to OA, increasing water temperature, and ecosystem shifts have the potential to impede adaptive responses. Monitoring walrus diets in conjunction with estimates of body condition and productivity will be useful to track potential changes in the prey base and subsequent effects on walrus. Limiting the potential impacts of activities that disturb the ocean floor (trawl fisheries, oil and gas well discharges, etc.) and affect walrus prey may also become important.

### Human adaptation

Many Alaskan and Chukotkan coastal communities continue to be closely tied to the Pacific walrus in many ways (EWC 2003; Oozeva et al. 2004; Metcalf and Robards 2008; Zdor et al. 2010). Changes in walrus ecology and population size have the potential to greatly impact these people (Hovelsrud et al. 2008; Metcalf and Robards 2008; Hill 2011). Climate change adaptation in this arena should focus on facilitating walrus hunting communities not only in terms of food security but in the preservation of opportunities to participate in the subsistence hunting culture. An example of one effort is the production of a walrus hunting video by the EWC and USFWS designed to familiarize and train younger hunters. Another facilitative program is the web-based Sea Ice for Walrus Outlook (http://www.arcus.org/search/siwo). This program provides weekly updates on ice distribution and 5- to 10-day forecasts of sea ice and weather conditions to assist walrus hunters and other community members. In addition, the EWC began an annual post-hunt survey of its Commissioners in 2006 that provides information on habitat changes and walrus behavior, physical condition, abundance, etc. that informs management programs. Furthermore, changes in the walrus co-management program, including management before depletion, as suggested by Metcalf and Robards (2008) and Robards et al. (2009) may also be helpful. Facilitating the management of coastal haulouts by local communities (Garlich-Miller 2012) not only empowers the community but also raises awareness of the importance of walrus to younger community members and provides a lesson in cultural tenets that view walrus as a valuable and respected member of the local community (J. G. MacCracken, pers. obs.).

Contaminant levels in the Arctic may increase due to climate change through increased mobilization, temperature accelerated chemical reactions, exposure of buried wastes due to coastal erosion and contaminants stored in the ice pack, permafrost, and vegetation, industrial accidents, and an overall increase in human activities, etc. (USFWS 2011). The effects of future contaminant levels on the Pacific walrus are unpredictable; however, the consumption of contaminated walrus is of concern to walrus hunting communities and a program of systematic testing of marine mammals is needed.

Pacific walrus face many challenges associated with a warming climate. Palaeological evidence suggests that they survived past warming events in situ or through migration to Arctic sea-ice refugia (Dyke et al. 1999; Jay et al. 2011). Two factors may distinguish the current warming event from those of the past: (1) the rate of change has been stated to be greater than in the past, and (2) there are also several additional, cumulative anthropogenic stressors. The first assertion may no longer be valid (Brigham-Grette 2009; Hof et al. 2011; Williams et al. 2011) and the latter has yet to fully materialize. Several stressors are occurring now (Wassmann et al. 2011) and will likely intensify in the future; other stressors may be occurring, but evidence is weak or nonexistent; and some stressors will not occur at significant intensity until sometime in the future (Ridgwell et al. 2009; Sigler et al. 2011).

High levels of greenhouse gas emissions are the ultimate cause of the changes that are impacting Pacific walruses. Regardless of how the climate changes in the future, programs that reduce the probability of large mortality events at fall coastal haulouts need to be continued, enhanced, and expanded. In addition, harvests need to continue to be closely monitored, struck and lost rates reduced, and methods to reliably assess the sustainability of the harvest developed. For impacts related to stressors that have yet to materialize, for example, OA, I have identified plausible scenarios that can guide monitoring and research programs and some potential mitigation actions.
